# Target volume coronary MRA revisited: usefulness of non-rigid reregistration of multi-frame 3D MRA acquisitions at 3T

**DOI:** 10.1186/1532-429X-17-S1-O51

**Published:** 2015-02-03

**Authors:** Masaki Ishida, Shinichi Takase, Ryohei Nakayama, Katsuhiro Inoue, Yoshitaka Goto, Yasutaka Ichikawa, Kakuya Kitagawa, Hajime Sakuma

**Affiliations:** 1 Radiology, Mie University Hospital, Tsu, Mie, Japan

## Background

Free-breathing whole-heart coronary MR angiography (MRA) is an established method that can visualize all coronary arteries within a single acquisition. However, a long acquisition time and suboptimal arterial signal due to thick SLAB are major limitations of 3T gradient-echo whole-heart coronary MRA without contrast. Alternatively, target-volume coronary MRA can be used to visualize coronary arteries within a predefined target volume within a shorter acquisition time. In addition, relatively small SLAB volume of this approach permits acquisitions of multi-frame 3D data without prolonging scan duration. Recently, non-rigid image registration has been emerged as a technique which can merge images and improve SNR and CNR. The purpose of this study was to develop a new technique to obtain high quality free-breathing target-volume coronary MRA with shorter acquisition time by employing multi-frame 3D acquisitions and non-rigid image registration.

## Methods

Six healthy volunteers underwent target volume coronary MRA and whole heart coronary MRA by using a TFE sequence with T2 preparation and fat saturation at 3T. For target volume coronary MRA, three successive 3D datasets were acquired separately for RCA and LCA during diastole (SNSE factor= 3; acquisition duration per cardiac cycle =30ms for RCA, 50ms for LCA; navigator gating window = 3mm; resolution =1.3x1.3x3.0 mm; slab thickness=4.5cm). Three-point planning system was used to define the imaging plane. Target volume MRA images were merged by using a non-rigid image registration technique optimized for coronary MRA. Two blinded reviewers determined SNR, CNR and a subjective quality score. Scan times for target-volume and whole-heart coronary MRA were compared for each volunteer.

## Results

**Figure 1:**
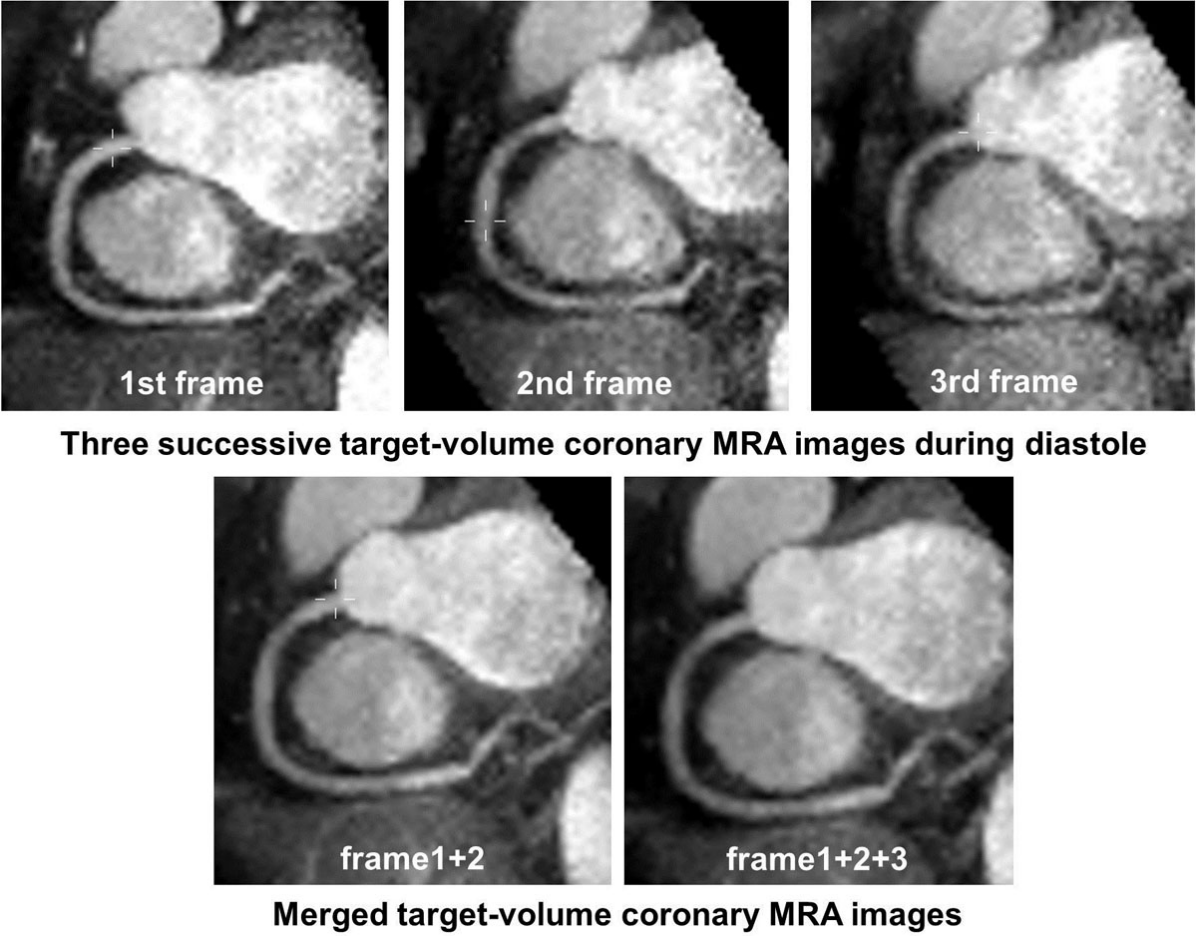
Target-volume coronary MRA for RCA. Three successive coronary MRA images during diastole (top) and merged coronary MRA images (bottom).

Target-volume coronary MRA successfully evaluated all segments of the coronary arteries in 6 volunteers (RCA#1-3, LMT#5, LAD#6-9, LCX #11-13) except for one distal LCx #13 segment in one case. Among 3 different frames in cardiac cycle, both SNR and CNR were the highest in the first frame (Table 1). When comparing the merged coronary MRA generated by non-rigid registration with the first-frame coronary MRA, SNR was continuously improved as the number of superposition increases, while CNR plateaued when the number of superposition exceeded two (Table, Figure 1). Subjective image quality score was substantially greater for merged coronary MRA than for the first-frame only coronary MRA (RCA 3.7±0.4 vs 3.1±0.8, p=0.10; LAD 3.9±0.2 vs 3.2±0.4, p=0.06; LCx 3.5±0.5 vs 2.9±0.6, p=0.06). Effective scan time for target-volume coronary MRA (416±81s) to cover all coronary arteries was significantly shorter than that of whole-heart coronary MRA (796±351s, p<0.04).

**Table 1:** SNR and CNR of coronary artery in target-volume coronary MRA.

	1st frame	2nd frame	3rd frame	Merged image (frame 1+2)	Merged image (frame 1+2+3)	p
SNR rca	13.2±4.4	9.8±3.7*	7.9±2.8*	16.7±6.7*	17.3±5.4*	* p<0.05

SNR lmt	14.5±3.5	13.4±3.6	10.8±2.9*	17.4±6.0*	19.2±4.1*	* p<0.05

SNR lad	13.7±2.3	13.7±1.9	11.5±3.2	17.2±3.7*	19.5±3.7*	* p<0.05

SNR lcx	13.1±3.5	11.5±3.8	10.0±3.8*	16.1±4.6*	17.1±5.2*	* p<0.05

CNR rca	6.8±3.0	3.2±2.3*	1.7±1.6*	7.9±5.0	7.6±4.5	* p<0.05

CNR lmt	8.1±2.0	6.8±2.2*	4.5±1.9**	8.6±4.0*	9.4±2.7*	* p<0.05, ** p<0.001

CNR lad	6.3±1.7	5.1±1.2	2.6±0.5*	7.1±2.5	7.1±2.3	* p<0.05

CNR lcx	6.8±2.5	4.9±2.8	3.8±2.5	7.4±3.1	7.4±3.8	* p<0.05

*, **: p value against 1st frame

## Conclusions

Multi-frame 3D acquisitions and non-rigid image reregistration allow for acquisition of free-breathing target volume 3T coronary MRA with the image quality that is superior to the single-frame acquisition, within a significantly shorter acquisition time compared to whole heart coronary MRA.

## Funding

N/A.

